# Supplementary material for: Predicting the effect of habitat modification on networks of interacting species
Source: Nat Commun. 2017 Oct 6;8:792. doi: 10.1038/s41467-017-00913-w (PMC5630616; doi:10.1038/s41467-017-00913-w)
Supplement: Supplementary file 1 — Supplementary Information [file 41467_2017_913_MOESM1_ESM.pdf]

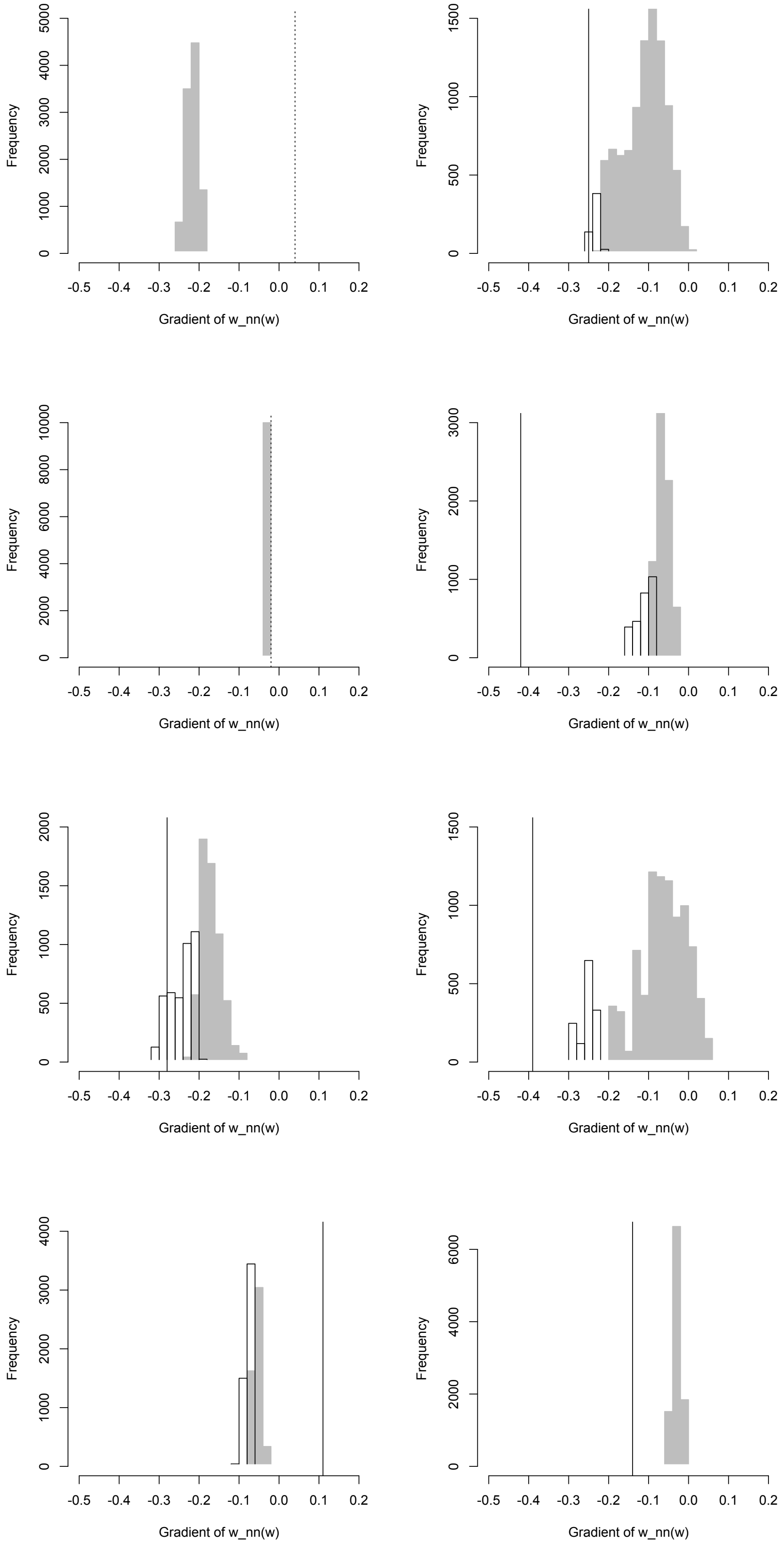

Supplementary Figure 1: Interaction assortativity and disassortativity.

Supplementary Figure 1: Interaction assortativity and disassortativity. A group of networks is interaction assortative if the gradient of $w_{\text{nn}}(w) > 0$ and interaction disassortative if the gradient of $w_{\text{nn}}(w) < 0$ (Eqn S21). Top-to-bottom: Ecuador {forest, coffee, abcoffee} (left) and {pasture, rice} (right); Indonesia {more forested} (left) and {less forested} (right); Swiss meadow {25m, 50m, 100m} (left) and {ECA} (right); Swiss lowland {most forested} (left) and {isolated} (right). The left column is representative of unmodified habitat types and the right column of modified habitat types. Vertical lines indicate the observed gradient of $w_{\text{nn}}(w)$ and is solid if significant (linear regression, p-value < 0.05) and dotted if not significant. Histogram bars indicate frequencies when parasitoid species rank-order preferences are maintained from the paired group of networks: open bars indicate that the gradient of $w_{\text{nn}}(w)$ is significant and filled bars indicate that it is not (for 10,000 realisations).

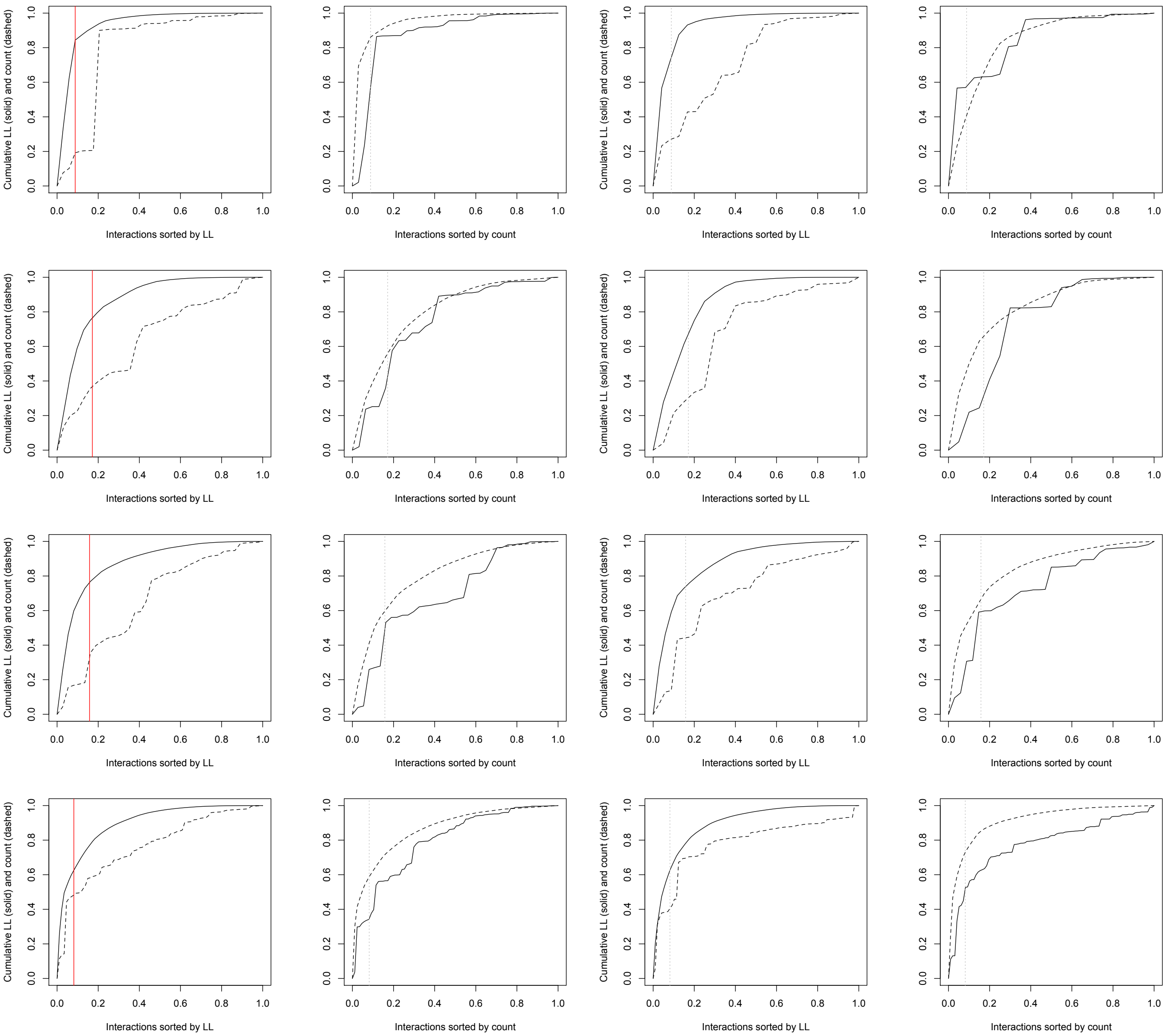

Supplementary Figure 2: Relative importance of interactions measured using log-likelihood (LL). Top-to-bottom: Ecuador {pasture, rice} (left-two columns) and {forest, coffee, abcoffee} (right-two columns); Indonesia {less forested} (left-two columns) and {more forested} (right-two columns); Swiss meadow {ECA} (left-two columns) and {25m, 50m, 100m} (right-two columns); Swiss lowland {isolated} (left-two columns) and {most forested} (right-two columns). Vertical solid (red) line indicates the cut-off for interactions hardcoded in the specified preferences model used to predict counts in the group associated with the left-two columns using data in the group associated with the right-two columns; vertical dotted (grey) line is at the same position but the identity of interactions may differ. Influential interactions to be hardcoded were determined by visually identifying the point at which the curve of cumulative LL begins to saturate and, therefore, the marginal LL of each additional interaction begins to decrease. Increasing or decreasing the number of hardcoded interactions changes model goodness-of-fit in proportion to the cumulative LL curve.

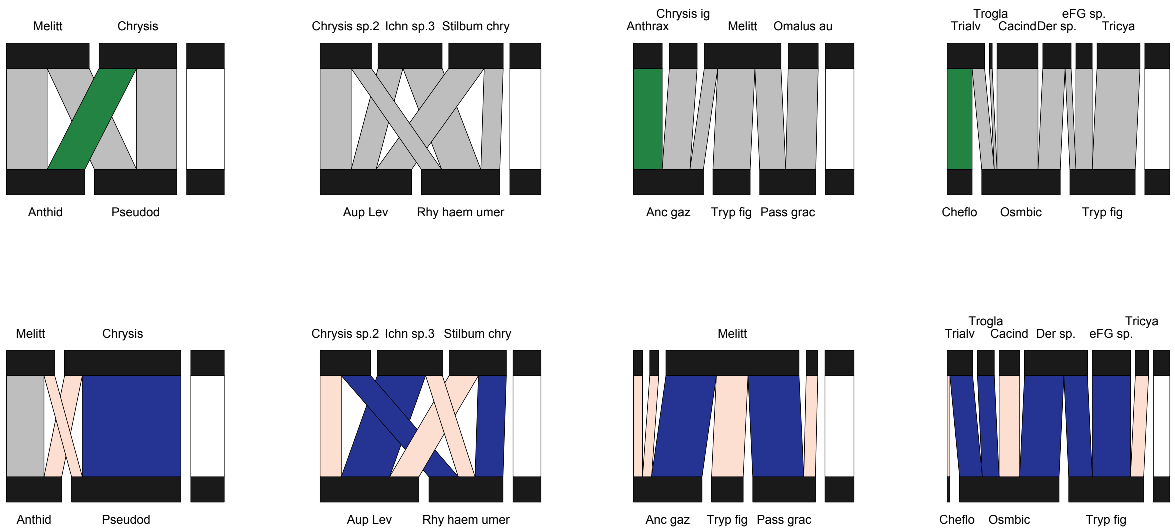

Supplementary Figure 3: Preference values of hardcoded interactions in the specified preferences model. Left-to-right: Ecuador {forest, coffee, abcoffee} (top) and {pasture, rice} (bottom); Indonesia {more forested} (top) and {less forested} (bottom); Swiss meadow {25m, 50m, 100m} (top) and {ECA} (bottom); Swiss lowland {most forested} (top) and {isolated} (bottom). The top row is representative of unmodified habitat types and the bottom row of modified habitat types. In each subfigure, top bars represent parasitoid species and bottom bars represent host species; incumbent interactions are in grey and mass action switches in green (top); significant increases in preference value in modified habitat types are coloured blue and significant decreases are coloured light red (bottom). The open bar in each subfigure indicates the interaction width that is consistent with mass action (i.e., with preference value equal to one). Full names of host and parasitoid species for Ecuador are Anthid: *Anthidium* sp.; Pseudod: *Pseudodynerus* sp.; Chrysis: *Chrysis* sp.; and Melitt: *Melittobia acasta* (the interaction between Anthid and Melitt is not included in the specified preferences model, but is included here for completeness). Indonesia, Aup Lev: *Auplopus levicarinatus*; Rhy haem umer: *Rhynchium haemorrhoidale umeroater*; Chrysis sp.2: *Chrysis* sp.2; Ich sp.3: *Ichneumonidae* sp.3; and Stilbum chry: *Stilbum chrysochephalum*. Swiss meadow, Anc gaz: *Ancistrocerus gazella*; Pass grac: *Passaloecus gracilis*; Tryp fig: *Trypoxylon figulus*; Anthrax: *Anthrax anthrax*; Chrysis ig: *Chrysis ignita*; Melitt: *Melittobia acasta*; and Omalus au: *Omalus auratus*. Swiss lowland, Cheflo: *Chelostoma florisomne*; Osmbic: *Osmia bicornis* (rufa); Tryp fig: *Trypoxylon figulus*; Cacind: *Cacoxenus indagator*; Der sp.:*Dermestidae* sp.; eFG sp.: undetermined; Trialv: *Trichodes alvearius*; Tricya: *Trichrysis cyanea*; and Trogla: *Trogoderma glabrum*.

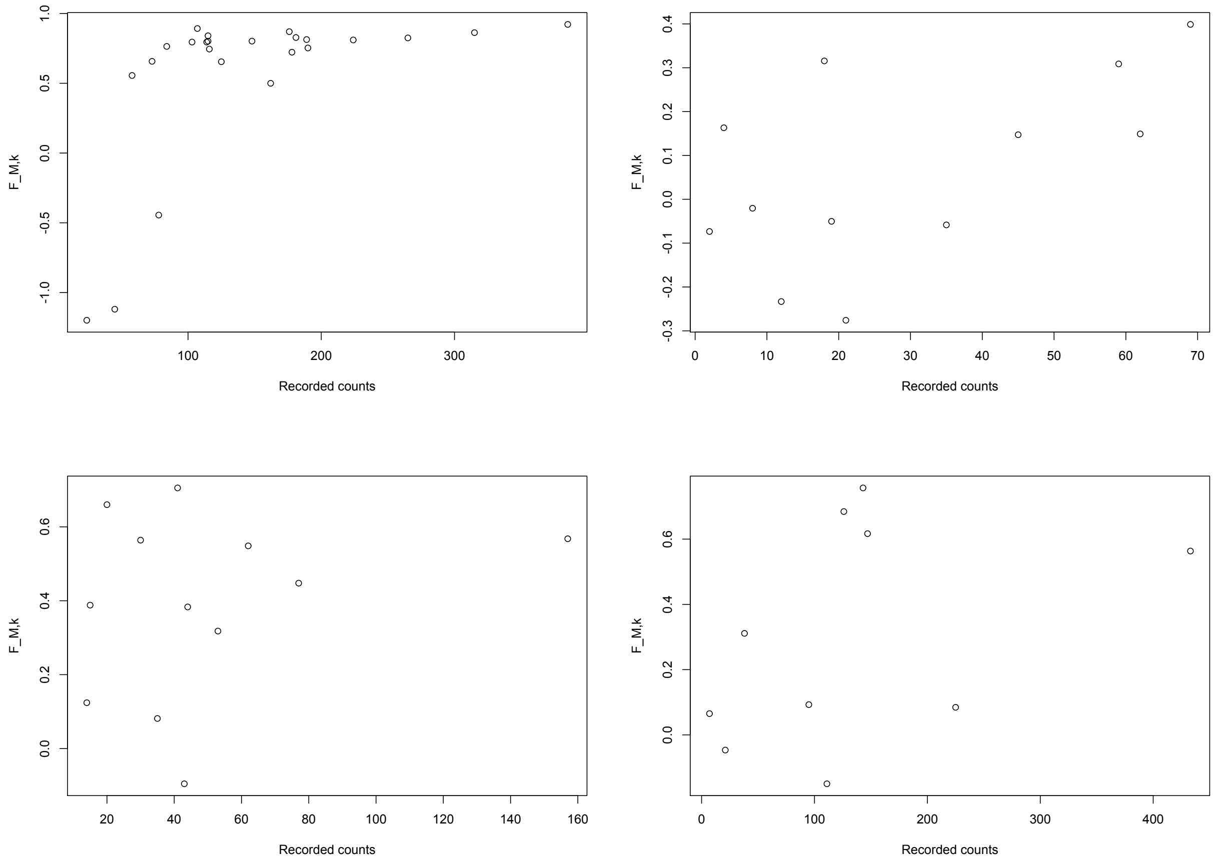

Supplementary Figure 4: Performance of the random encounter model at predicting weighted network structure at individual field sites in modified habitat types as a function of recorded counts. Top row (left-to-right): Ecuador {pasture, rice} and Indonesia {less forested}; bottom row (left-to-right): Swiss meadow {ECA} and Swiss lowland {isolated}. Fit is relative to a null model in which all non-forbidden interactions are equally likely to be observed, $\mathcal{F}_{M,k} = \frac{\mathcal{L}_{\text{null},k} - \mathcal{L}_{M,k}}{\mathcal{L}_{\text{null},k}}$ (Eqn S25). $\mathcal{F}_{M,k} = 1$ if model M completely explains the distribution of recorded interaction counts at field site k; $\mathcal{F}_{M,k} = 0$ if it performs the same as the null model; and $\mathcal{F}_{M,k} < 0$ if it performs worse than the null model. Notice that the random encounter model performed less well at field sites with very few recorded counts. This was due to the limited possibility for non-random and ecologically meaningful weighted structure to be observed in networks built using small amounts of interaction data.

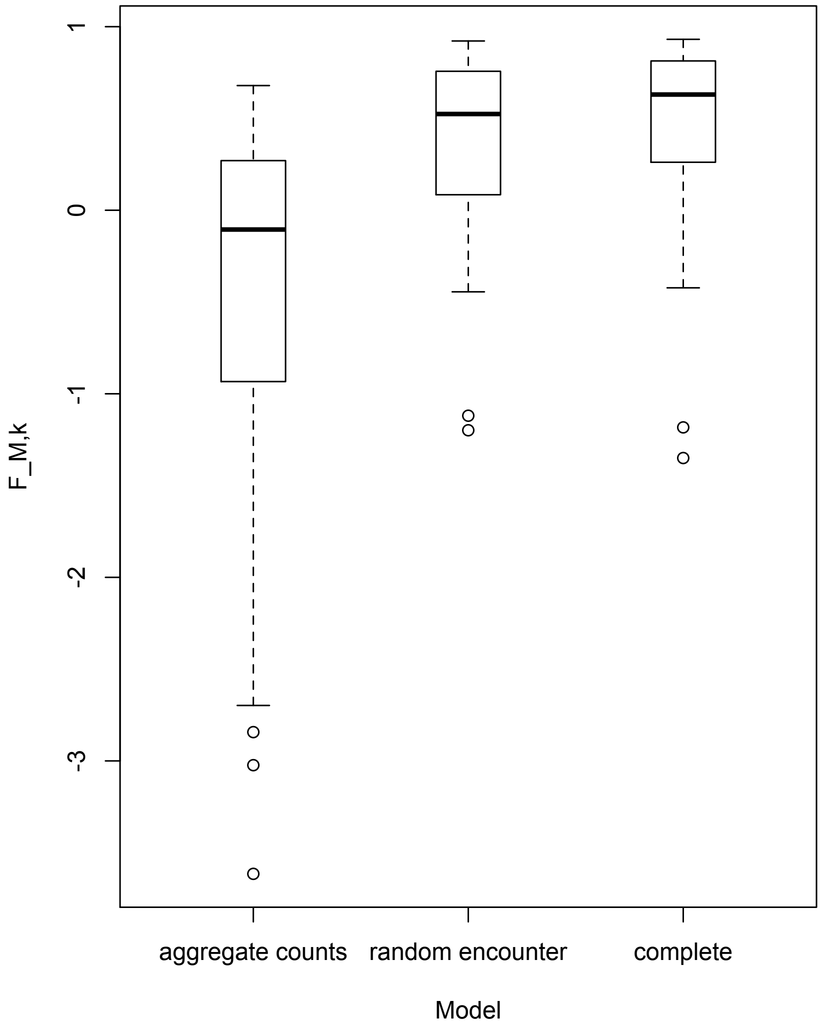

Supplementary Figure 5: Performance of three models at predicting weighted network structure at individual field sites in modified habitat types for all four data sets. Fit is relative to a null model in which all non-forbidden interactions are equally likely to be observed, $\mathcal{F}_{M,k} = \frac{\mathcal{L}_{\text{null},k} - \mathcal{L}_{M,k}}{\mathcal{L}_{\text{null},k}}$ (Eqn S25). $\mathcal{F}_{M,k} = 1$ if model M completely explains the distribution of recorded interaction counts at field site k; $\mathcal{F}_{M,k} = 0$ if it performs the same as the null model; and $\mathcal{F}_{M,k} < 0$ if it performs worse than the null model. Box plots for the alternative preferences, correlated preferences and specified preferences models are similar to the box plots for the random encounter and complete characterisation models, so have been omitted for clarity. Box plot whiskers extend to the lowest datum within 1.5 $\times$ interquartile range of the lower quartile and the highest datum within 1.5 $\times$ interquartile range of the upper quartile, with outliers identified by open circles.

Supplementary Table 1: Properties of paired groups of networks. Number of parasitoid (P) and host species (H); habitat complexity; consumer-resource ratio; suggested state of habitat modification, modified (mod) or unmodified (unmod); maximum likelihood parameters for mass action ($\hat{\alpha}$ and $\hat{\beta}$) and resulting log-likelihood ($\mathcal{L}_{\text{ma}}$); log-likelihood when $\alpha = \beta = 1$; and group-level deviation of weighted network structure from mass action, $\mathcal{B} = \frac{\mathcal{L}_{\text{ma}} - \mathcal{L}_{\text{complete}}}{\mathcal{L}_{\text{ma}}} \in [0, 1]$.

Data set	Species richness	Network group	Common species	Habitat complexity	Con-res ratio	State	$\hat{\alpha}$	$\hat{\beta}$	$\mathcal{L}_{\text{ma}}$	$\mathcal{L}_{\alpha=\beta=1}$	$\mathcal{B}$
Ecuador	P: 9<br>H: 33	{forest, coffee, abcoffee}	P: 7	forested	low (0.08)	unmod	1	0.85	−287	−288	0.01
		{pasture, rice}	H: 30	open	high (0.22)	mod	0.8	0.55	−1554	−1778	0.21
		{pasture}	P: 6	open	high (0.25)	mod	0.75	0.55	−881	−1059	0.15
		{rice}	H: 23	open	high (0.18)	mod	0.85	0.6	−541	−581	0.24
Indonesia	P: 25	{more forested}	P: 14	forested	low (0.04)	unmod	0.85	0.85	−238	−240	0.07
	H: 14	{less forested}	H: 12	open	low (0.05)	mod	0.65	0.8	−260	−263	0.05
Swiss meadow	P: 16	{25m, 50m, 100m}	P: 13	open	low (0.12)	unmod	1.35	1.2	−381	−385	0.07
	H: 20	{ECA}	H: 18	open	high (0.20)	mod	0.85	1	−231	−234	0.18
Swiss lowland	P: 61<br>H: 49	{most forested}	P: 24	forested	high (0.19)	unmod	0.65	0.9	−2427	−2501	0.31
		{isolated}	H: 28	open	high (0.16)	mod	0.85	0.75	−824	−851	0.30
		{adjacent}	P: 29	forested	high (0.23)	unmod	0.55	0.65	−3208	−3580	0.37
		{connected}	H: 36	forested	high (0.21)	unmod	0.7	0.75	−2271	−2348	0.27

Supplementary Table 2: Significance of changes in interaction preferences between paired groups of networks. Total number of interactions in second-named group in prediction direction, E; separated into incumbent interactions and switches, where sig, sig ↑ and sig ↓ indicate the number of significant changes in interaction preference all together, that increase and that decrease, respectively (10,000 realisations and significance level is p-value < 0.01). Right-most column is the probability of obtaining more switches than observed when networks are grouped randomly and not according to habitat type (10,000 realisations).

			Incumbent				Switches				
Data set	Prediction direction	E	Tot	sig	sig ↑	sig ↓	Tot	sig	sig ↑	sig ↓	prob
Ecuador	{forest, coffee, abcoffee} to {pasture, rice}	34	15	9	6	3	19	7	3	4	7.0E-4
				60%	40%	20%		37%	15%	12%	
	reverse	28	15	5	1	4	13	4	0	4	0.26
				34%	7%	27%		30%	0%	30%	
	{pasture} to {rice}	25	19	2	2	0	6	2	1	1	0.59
				10%	10%	0%		34%	17%	17%	
	reverse	25	19	8	5	3	6	3	2	1	0.59
				42%	26%	16%		50%	33%	17%	
Indonesia	{more forested} to {less forested}	35	20	7	5	2	15	0	0	0	0.033
				35%	25%	10%		0%	0%	0%	
	reverse	22	20	4	1	3	2	2	0	2	0.97
				20%	5%	15%		100%	0%	100%	
Swiss meadow	{25m, 50m, 100m} to {ECA}	38	21	9	6	3	17	5	2	3	1.7E-3
				42%	28%	14%		29%	12%	17%	
	reverse	38	21	6	4	2	17	4	0	4	0.15
				28%	19%	9%		24%	0%	24%	
Swiss lowland	{most forested} to {isolated}	98	59	25	16	9	39	8	0	8	0.88
				42%	27%	15%		20%	0%	20%	
	reverse	112	59	19	7	12	53	17	0	17	0.41
				32%	12%	20%		32%	0%	32%	
	{adjacent} to {connected}	130	78	25	14	11	52	8	1	7	0.53
				32%	18%	14%		15%	2%	13%	
	reverse	130	78	29	13	16	52	12	5	7	0.53
				37%	17%	20%		23%	10%	13%	

Supplementary Table 3: Log-likelihood of models based on mass action. Log-likelihoods calculated at the group level (Eqn S3 for all k-indices, and, therefore, all field sites in a group of networks). In prediction direction, the first listed group is the calibration group and the second listed group is the test group. Log-likelihood calculated for the random encounter model (re), alternative preferences model with mass action switches (alt, maS) and binomial method switches (alt, bmS), correlated preferences model (corr, with maximum likelihood scaling parameter $\hat{\delta}$ in parenthesis; and with $\delta = 1$), two specified preferences models (spec, with non-hardcoded interaction preferences taken from the alternative preferences model; and spec2, with non-hardcoded interaction preferences set to 1), specified preferences model combined with correlated preferences model (spec+corr, with maximum likelihood scaling parameter $\hat{\delta}$ in parenthesis; and with $\delta = 1$), and complete characterisation model (comp). The specified preferences models are only relevant for prediction between groups with different habitat complexity and/or consumer-resource ratio. Less-negative values indicate better fit.

Data set	Prediction direction	$\mathcal{L}_{\text{re}}$	$\mathcal{L}_{\text{alt, maS}}$	$\mathcal{L}_{\text{alt, bmS}}$	$\mathcal{L}_{\text{corr}}$ $(\hat{\delta})$	$\delta = 1$	$\mathcal{L}_{\text{spec}}$	$\mathcal{L}_{\text{spec2}}$	$\mathcal{L}_{\text{spec+corr}}$ $(\hat{\delta})$	$\delta = 1$	$\mathcal{L}_{\text{comp}}$
Ecuador	{forest, coffee, abcoffee} to {pasture, rice}	−1554	−1550	−1584	−1409 (2.4)	−1456	−1264	−1265	−1258 (0.6)	−1260	−1220
	reverse	−287	−371	−473	−286 (0.2)	−300	−307	−286	−285 (0.3)	−293	−283
	{pasture} to {rice}	−541	−407	−408	−397 (0.7)	−428	na	na	na	na	−375
	reverse	−881	−744	−744	−757 (0.9)	−758	na	na	na	na	−715
Indonesia	{more forested} to {less forested}	−260	−265	−281	−254 (0.6)	−257	−249	−255	−249 (0.8)	−250	−245
	reverse	−238	−237	−237	−230 (0.5)	−242	−222	−231	−222 (1.1)	−222	−221
Swiss meadow	{25m, 50m, 100m} to {ECA}	−231	−244	−291	−222 (0.6)	−226	−202	−204	−199 (0.6)	−202	−189
	reverse	−381	−410	−512	−368 (0.5)	−379	−371	−371	−366 (0.7)	−367	−354
Swiss lowland	{most forested} to {isolated}	−824	−789	−902	−674 (0.6)	−710	−662	−778	−639 (0.7)	−665	−574
	reverse	−2427	−2098	−2131	−1913 (0.9)	−1923	−1877	−2273	−1843 (0.9)	−1849	−1657
	{adjacent} to {connected}	−2271	−1899	−1916	−1796 (0.6)	−1939	na	na	na	na	−1643
	reverse	−3208	−2388	−2369	−2368 (1.0)	−2368	na	na	na	na	−2004

SUPPLEMENTARY NOTE 1: Host-parasitoid interaction networks

We analysed the weighted interaction network structure of cavity-nesting bees, wasps and their parasitoid consumers collected in four independent studies* where both modified and unmodified habitat types were sampled. The four studies involve similar guilds of interacting parasitoid and host species, and together comprise two tropical regions, Ecuador[1] and Indonesia[2], and two temperate regions, meadows in Switzerland[3] and lowland plains in Switzerland[4].

Networks were constructed from data on hosts collected in trap nests. Each host was reared and identified, and any emerging parasitoid was also identified. The emergence of a parasitoid defines a successful parasitism event, which was recorded as a *count* between the identified parasitoid and host species. The weight of an interaction in a network is the total number of recorded counts between two species at a given field site. If no parasitoid emerged following the rearing of a host, then the event was categorised as *unparasitised*, even though the host may have been parasitised but was able to prevent the parasitoid from completing its adult stage. Therefore in addition to multiple networks built from interaction data collected at different field sites, each data set also included a record of the number of parasitised and unparasitised individuals for each host species.

*We required that data sets satisfy three criteria for inclusion in our analysis: i) the lower trophic level involves cavity-nesting insects, so that the method of data collection is similar among data sets; ii) networks were sampled from what could broadly be considered "modified" and "unmodified" habitats, so that we could assess the effect of habitat modification on weighted network structure; and iii) more than ten networks were sampled from field sites in each different habitat type, so that we could assess changes in weighted network structure both at individual field sites and at a group level (i.e., across multiple field sites at once).

Network properties, grouping networks and habitat complexity

We are interested in predicting weighted network structure at field sites in human-modified habitats (mainly open habitats) using models calibrated with data from relatively unmodified habitats (mainly forested habitats). We grouped networks in each data set by habitat type and used metadata to identify the level of habitat complexity within each group as either *forested* or *open*.

Ecuador

Data include 48 networks sampled from five habitat types: forest (6 networks); shade-grown coffee agroforest (which we refer to as *coffee*, 12); abandoned coffee agroforest (*abcoffee*, 6); pasture (12); and rice (12). Total species richness across all networks is 9 parasitoid species and 33 host species; and the total number of recorded successful parasitism events is 4095 (forest, 88; coffee, 322; abcoffee, 113; pasture, 2162; and rice, 1410).

There is a clear difference in average tree species richness across field sites among the five habitat types: forest, 21.3; coffee, 7.5; abcoffee, 17; pasture, 0; and rice, 0. Similarly, there is a clear difference in average measured light intensity at ground level (units of lux, where lower values indicate greater forest coverage as less light reaches ground level): forest, 86.1; coffee, 82.9; abcoffee, 99.3; pasture, 668.2; and rice, 711.1. Based on these differences, the group of networks {forest, coffee, abcoffee} contains networks sampled from high complexity, forested habitats (24 networks); and {pasture, rice} contains networks sampled from low complexity, open habitats (24 networks). There were also sufficient data to consider two groups of open networks: {pasture} and {rice}.

Indonesia

Data include 24 networks sampled from the same agroforest. We grouped networks according to canopy coverage at their respective field sites: more forested (12 networks) and less forested (12). Average measured light intensity is 152.5 $\pm$ 70.0 lux (mean $\pm$ standard deviation) for {more forested}, while it is 591.2 $\pm$ 215.1 lux for {less forested}. Total species richness across all networks is 25 parasitoid species and 14 host species; and the total number of recorded successful parasitism events is 731 (more forested, 356; and less forested, 375).

Swiss meadow

Data include 47 networks sampled from two habitat types: restored meadow (classified as an ecological compensation area, ECA, 13 networks); and intensively managed meadows at distances 25m (11 networks), 50m (12) and 100m (11) from the nearest ECA. Total species richness across all networks is 16 parasitoid species and 20 host species; and the total number of recorded successful parasitism events is 1351 (ECA, 604; 25m, 295; 50m, 222; and 100m, 230).

We considered two groups: {ECA} (13 networks) and {25m, 50m, 100m} (34 networks). Average plant species richness is different between the habitat types: ECA, 32.4; and 25m, 16.9; 50m, 18.0; and 100m, 18.8. Interaction count is much higher among ECA networks (total count was 604 in {ECA} and 747 in {25m, 50m, 100m}), so having different numbers of networks between the two groups was not an issue. Some of the sampled ECAs had one or very few fruit trees, while almost no intensively managed meadows had trees. Both groups therefore contain networks sampled from low complexity, open habitats.

Swiss lowland

Data include 30 networks sampled from three habitat types: adjacent to forest (which we refer to as *adjacent*, 10 networks); located at a distance of 100m–200m from the nearest forest, but still connected to the forest by woody elements such as hedgerows or rows of single trees (*connected*, 10); and, finally, located at least 100m away from any woody habitat including forests, hedgerows and orchards (*isolated*, 10). Total species richness across all networks is 61 parasitoid species and 49 host species; and the total number of recorded successful parasitism events is 8871 (adjacent, 4395; connected, 3189; and isolated, 1387).

In the original study[4], field sites were standardised by planting, at each site, an 18m-long row of young cherry trees on permanent grassland. The fraction of woody habitat within 500m of each site was similar among habitat types: adjacent, 0.27 $\pm$ 0.23 (mean $\pm$ standard deviation); connected, 0.28 $\pm$ 0.21; and isolated, 0.29 $\pm$ 0.24. (A circular area with radius of 500m was chosen because this distance roughly matched the activity range of the studied organisms). Despite similar fractions of woody habitat around sites, we consider the isolated habitats to be low complexity, open habitats because there were no paths from any woody habitat to the grassland sites where sampling took place. By contrast, we consider adjacent and connected habitats to be high complexity, forested habitats because there were direct paths from forest to grassland, so the makeup of habitat within the 500m radius was likely to influence parasitoid-host communities at field sites. Furthermore, communities in adjacent and connected habitats were found to respond similarly to their (lower) level of isolation from forest compared to isolated habitats[4]. Consequently, to increase the difference in habitat complexity between open and forested habitats in our analysis, we grouped the five networks from adjacent habitats with the highest fraction of woody habitat with

the likewise five networks from connected habitats. This group of the ten most-forested networks has 0.45 ± 0.16 fraction of woody habitat within the 500m radius.

With these data, we considered two groups to assess the effect of habitat modification: {ten most-forested from adjacent and connected} contains networks sampled from high complexity, forested habitats (10 networks each); and {isolated} contains networks sampled from low complexity, open habitats (10 networks). There were also sufficient data for two groups of forested networks: {adjacent} and {connected}.

Consumer-resource ratio and parasitism rate

In addition to habitat complexity, we calculated the consumer-resource ratio for each group of networks, which, for these data, is equivalent to average parasitism rate. We defined consumer-resource ratio as the total number of successful parasitism events across all species divided by the total number of parasitised and unparasitised hosts collected in the field. This measure indicates how easily parasitoids are able to locate their hosts in particular habitat types, and we labelled groups as being associated with either *low* or *high* consumer-resource ratio.

Ecuador

The group of forested networks {forest, coffee, abcoffee} has a consumer-resource ratio of 0.08, whereas the group of open networks {pasture, rice} has a higher consumer-resource ratio of 0.22. Therefore, {forest, coffee, abcoffee} contains networks sampled from habitats with low consumer-resource ratio (24 networks); whereas {pasture, rice} contains networks sampled from habitats with high consumer-resource ratio (24 networks). Separately, {pasture} has a consumer-resource

ratio of 0.25 and {rice} has 0.18, so both groups contain networks sampled from habitats with high consumer-resource ratio.

Indonesia

The group of forested networks {more forested} has a consumer-resource ratio of 0.04, and the group of open networks {less forested} has a similar consumer-resource ratio of 0.05. Therefore, both groups contain (12) networks sampled from habitats with low consumer-resource ratio.

Swiss meadow

The group of {25m, 50m, 100m} networks has a consumer-resource ratio of 0.12, whereas {ECA} has 0.20. Although both groups contain networks sampled from open habitats, there is a clear difference in consumer-resource ratio: {25m, 50m, 100m} contains networks sampled from habitats with low consumer-resource ratio (34 networks); whereas {ECA} contains networks sampled from habitats with high consumer-resource ratio (13 networks).

Swiss lowland

The three groups of forested networks {adjacent}, {connected} and {ten most-forested from adjacent and connected} have high consumer-resource ratios of 0.23, 0.21 and 0.19, respectively. The group of open networks {isolated} has a lower but still relatively high consumer-resource ratio of 0.16 (compared to groups in the other three data sets). Therefore, all groups contain (10) networks sampled from habitats with high consumer-resource ratio.

Habitat modification and changes in habitat complexity and consumer-resource ratio

We compiled a total of 12 groups of networks with sufficient data for analysis and identified two features with each group: habitat complexity (forested or open) and consumer-resource ratio (low or high) (see above and summarised in Supplementary Table 1). This enabled us to test predictions between similar habitat types (two groups in the same data set with the same combination of features) and different habitat types (two groups with a different combination) (see Fig. 1 in main text). As a direction for prediction, we focused on forested-to-open for the three data sets for which this was possible, and low-to-high for the Swiss meadow data set. We restricted our analysis to subsets of host and parasitoid species that are present in both, paired, groups of networks. However, all results are qualitatively similar if the complete set of species is considered instead (results not shown).

Taxonomic aggregation of networks

Our approach is based on food webs resolved to the level of individual species. At least in host-parasitoid systems, this is likely to be the most relevant level of analysis because of the intimate, co-evolved relationships between hosts and parasitoids, and their relatively high specificity. This is also the level of aggregation most frequently used in empirical network studies, although separation within species (e.g., according to genotypes) or aggregations of species are occasionally published. More importantly, species-level partitioning of host resources across parasitoids has been shown empirically to relate to functional properties such as attack rates and their stability[5], so there is an established relationship between network architecture with species as nodes and ecosystem processes. For larger community networks, particularly those that are more taxonomically and func-

tionally diverse[6], and which include a wider range of interactions types (e.g., predator-prey food webs), a definition of nodes in terms of aggregations of taxa may be equally or more illuminating about dynamics compared to an approach focused on species-level interactions. Nevertheless, the general approach and methods introduced in this study are applicable to taxonomically aggregated networks, but care must be taken that predictive models reflect ecological mechanisms and processes that are appropriate for the level of aggregation under investigation.

SUPPLEMENTARY NOTE 2: Interaction preferences

For predicting weighted network structure, the simplest model that separates changes in relative species abundance from other, potentially confounding changes is mass action. With mass action, the frequency of an interaction, and therefore the expected number of recorded counts, is proportional to the product of host and parasitoid species' abundances[7,8]. Ecological network data, however, rarely include independent measurements of species abundance or local population density. But given sufficient count data, we can estimate relative species abundances that are consistent with mass action[9]. Estimating relative species abundance from interaction data is an inverse problem. This is because observed measurements (counts) are used to infer information about a property of a physical system that has not been directly observed (abundances). We stress that these estimated abundances may be different from independent measurements because they represent idealised abundances that provide the closest agreement to data under the mass action hypothesis; they should therefore be considered effective or functional species abundances. Given a set of independently measured or estimated abundances, we define the preference of an interaction as the deviation of counts (successful parasitism events) from the expected number of counts according to mass action. Such preferences are, in principle, independent of individual species' local population density or abundance. We therefore suggest that it is more informative to compare interaction preferences between different field sites and habitat types than absolute numbers of recorded counts.

In this note, we present a likelihood-based method for determining interaction preferences for a group of networks. We show how to determine if interaction preferences between two groups of networks are significantly different and also provide a measure for the group-level deviation of weighted network structure from mass action.

Effective abundances

Host-parasitoid interaction networks are built from the number of successful parasitism events recorded between parasitoid and host species (see Supplementary Note 1). As such, the raw data of an interaction network does not, itself, contain independent measurements of species abundance. Here we describe our method for estimating relative species abundance from recorded count data.

Consider a group of networks. Assuming a general form of mass action, the recorded number of counts $B_{ijk} > 0$ between host species i and parasitoid species j at field site k can be written as

$$B_{ijk} \propto x_i^{\alpha} x_j^{\beta} \tag{S1}$$

where x_i and x_j are species' abundances to be estimated (note that Eqn S1 assumes that abundances hold across all networks in a group, which is a necessary assumption because there are often too few data in individual networks to determine non-trivial abundance estimates); and α and β are parameters that apply to all host and parasitoid species, respectively.

For $\alpha, \beta > 1$, the distribution of estimated abundances is compressed relative to the simplest version of mass action ($\alpha = \beta = 1$); while for $\alpha, \beta < 1$, the distribution is stretched. The situation when $\alpha, \beta < 1$ is similar to a Type II numerical response[10]. This is because species with relatively large estimated abundance are involved in fewer interactions compared to $\alpha = \beta = 1$ (more so than species with smaller estimated abundance), with the overall effect similar to a decelerating numerical response to prey density that ultimately saturates. By contrast, the situation when $\alpha, \beta > 1$ is similar to the initial stage of a Type III numerical response[10]. In this case, species with relatively large estimated abundance are involved in a greater number of interactions compared to $\alpha = \beta = 1$, with the overall effect similar to an accelerating numerical response to prey density.

Taking the natural logarithm of Eqn S1,

$$\ln(B_{ijk}) \propto \alpha \ln(x_i) + \beta \ln(x_j) \tag{S2}$$

For a given pair of α and β values, if the network ($k = 1$) or group of networks ($k > 1$) is sufficiently dense with interactions then we have a set of overdetermined equations involving only recorded counts, with one equation for each recorded B_{ijk}. This set of overdetermined equations can be solved to obtain estimates of x_i and x_j. In practice, we used the function *lsei*, which is part of the R package *limSolve*[11].

Likelihood function based on the multinomial distribution

We assume that the set of recorded counts in a group of networks follows a multinomial distribution[12]. With the multinomial distribution, each interaction has a distinct probability p_{ijk} of being recorded and the likelihood function is

$$L(p_{ijk}|B_{ijk}) = P(B_{ijk}|p_{ijk}) = \frac{(\sum_{ijk} B_{ijk})!}{\prod_i \prod_j \prod_k B_{ijk}!} \prod_i \prod_j \prod_k (p_{ijk})^{B_{ijk}} \tag{S3}$$

Under mass action, the likelihood for a pair of α and β values and their associated set of estimated species' abundances, x_i and x_j, given count data is

$$L(\alpha, \beta, x_i, x_j|B_{ijk}) = P(B_{ijk}|\alpha, \beta, x_i, x_j) = \frac{(\sum_{ijk} B_{ijk})!}{\prod_i \prod_j \prod_k B_{ijk}!} \prod_i \prod_j \prod_k \left(\frac{x_i x_j}{\sum_{ij} x_i x_j}\right)^{B_{ijk}} \tag{S4}$$

where we have set the probability of a given interaction as $p_{ijk} = f(\alpha, \beta) = \frac{x_i x_j}{\sum_{ij} x_i x_j}$, which is proportional to the product of interacting host and parasitoid species' abundances, as required. The log-likelihood is $\mathcal{L} = \ln(L)$. In practice, we calculated log-likelihoods using the function *dmultinomin*, which is part of the standard statistics package of R[11].

We chose this likelihood function because it describes how well a model is able to explain the recorded distribution of interaction counts among species at a field site. We can understand the multinomial distribution by considering a bag containing $\sum_{ijk} B_{ijk}$ balls, each representing a successful parasitism event. Balls are drawn one at a time from the bag and are assigned to pot B_{ijk} with probability $p_{ijk} = f(\alpha, \beta) = \frac{x_i x_j}{\sum_{ij} x_i x_j}$. The likelihood is the probability of generating an observed distribution of successful parasitism events—weighted network structure—given the estimated relative species abundance associated with a particular pair of α and β values.

Finding maximum likelihood estimate parameters

We used the above likelihood function to determine the most appropriate values of α and β on a group-by-group basis. For each group, we trialled combinations of $0.05 \leq \alpha \leq 2$ and $0.05 \leq \beta \leq 2$ in increments of 0.05 and recorded resulting log-likelihoods using Eqn S4. The pair of α and β values that resulted in the largest log-likelihood is the maximum likelihood estimate pair of parameters, $\hat{\alpha}$ and $\hat{\beta}$, and we denote their associated maximum likelihood estimate densities by $\hat{x}_i$ and $\hat{x}_j$ for host and parasitoid species, respectively. Maximum likelihood parameters for the 12 groups in our analysis are given in Supplementary Table 1, along with their resulting log-likelihoods and, for comparison, log-likelihoods when $\alpha = \beta = 1$.

We scaled maximum likelihood estimate abundances by a constant so that the expected number of counts following mass action is equal to the total number of recorded counts:

$$\hat{x}_i \leftarrow \left(\frac{\sum_{ijk} B_{ijk}}{\sum_{ij} \hat{x}_i \hat{x}_j} \right)^{\frac{1}{2}} \hat{x}_i \quad \text{and} \quad \hat{x}_j \leftarrow \left(\frac{\sum_{ijk} B_{ijk}}{\sum_{ij} \hat{x}_i \hat{x}_j} \right)^{\frac{1}{2}} \hat{x}_j \tag{S5}$$

Note that this transformation does not change corresponding log-likelihoods because the likelihood function, Eqn S4, is invariant to a linear multiplicative factor.

Calculating interaction preferences

We define interaction preferences at the level of an individual field site as

$$\gamma_{ijk} = \frac{B_{ijk}}{\hat{x}_i \hat{x}_j} \tag{S6}$$

where, as mentioned above, we assume that estimated species' abundances hold across all networks in a group. We obtain a two-dimensional preference matrix that is representative of the group by averaging across sites:

$$\gamma_{ij} = \frac{\sum_k \gamma_{ijk}}{\sum_k a_{ijk}} \tag{S7}$$

where $a_{ijk} = 1$ if $\gamma_{ijk} > 0$ and $a_{ijk} = 0$, otherwise.

In general, a value of $\gamma_{ij} > 1$ indicates an interaction with recorded count higher than that expected from mass action, so is considered a preferred interaction; $\gamma_{ij} < 1$ indicates an interaction with recorded count lower than that expected from mass action, so is considered a less-preferred interaction; and $\gamma_{ij} = 1$ indicates an interaction with recorded count equal to that expected from mass action. (Note that if the set of equations described by Eqn S2 is not overdetermined but underdetermined, then $\gamma_{ij} = 1$ for all i and j for which $\sum_k B_{ijk} > 0$, which indicates that an infinite number of solutions exist and, therefore, there is not enough information to infer non-trivial values for interaction preferences.)

This definition of interaction preferences (Eqn S7) extends the original definition[9] in two ways: i) parameters α and β (see Eqn S1) have been introduced where previously $\alpha = \beta = 1$ was implicitly assumed; and ii) interaction preferences are averaged across networks in a group, where previously only aggregated networks were considered (as data on individual networks were not available). As we discuss below, the particular values of α and β can be important. Also, although

Eqn S7 is applicable to single, aggregated networks, we recommend, if possible, analysing data as a group of individual, field site-specific networks to avoid mis-estimating relative species abundances and therefore interaction preferences, as discussed in the main text. (Indeed, Eqn S7 is applicable to single, field site-specific networks, but they must be sufficiently dense with interactions to avoid inferring trivial interaction preferences, as mentioned above).

Measuring the significance of changes in interaction preference

Each recorded interaction between a parasitoid and host species in a group of networks has an associated preference value (γ_{ij}, Eqn S7). This interaction preference indicates how the recorded number of successful parasitism events deviates from the expectation according to mass action. If we consider the same interaction in a different group of networks then we may obtain a different value for its preference. We can determine whether this new interaction preference is significantly different by comparing it, as a point estimate, to the distribution of preference values generated using data from the original group of networks. This procedure results in a p-value for each observed interaction that indicates the significance of any change in interaction preference between two groups of networks.

Consider two groups of networks containing a common subset of host and parasitoid species, Group 1 and Group 2, and we want to know whether the interaction preferences in Group 2 are significantly different from their respective values in Group 1. First, a set of synthetic preference values for each interaction is generated using the data of Group 1. There are four steps: i) a set of synthetic counts is drawn from a multinomial distribution with probabilities that are proportional to the relative distribution of recorded counts in Group 1, i.e., $p_{ijk} = \frac{B_{ijk}}{\sum_{ijk} B_{ijk}}$ in Eqn S3 (and the

total number of synthetic counts is the same as the total number of recorded counts in Group 1); ii) synthetic entries with no count are changed to one, and, to compensate for these additional counts, one count is removed from each of the largest synthetic counts in turn until the total number of synthetic counts equals the recorded number; iii) a set of synthetic interaction preferences is calculated (Eqn S7) from the set of synthetic counts using the same $\hat{\alpha}$ and $\hat{\beta}$ as for the recorded counts in Group 1; and iv) steps i–iii are repeated 10,000 times to generate a distribution of preference values for each observed interaction in Group 1.

For an interaction observed in both groups, which we refer to as an incumbent interaction, the significance of its (change in) preference in Group 2 can be measured by a p-value. If the preference in Group 2 is greater than the mean of the synthetic distribution (which is generated from data from Group 1 and approximately normally distributed), then the p-value is equal to the fraction of values in the distribution that is greater than the observed value in Group 2. In other words, the p-value is the probability of observing a preference value in Group 1 that is greater than the observed preference value in Group 2. Conversely, if the preference in Group 2 is lower than the mean of the synthetic distribution, then the p-value is equal to the fraction of values in the distribution that is lower than the observed value in Group 2; so the p-value is the probability of observing a preference value in Group 1 that is lower than the observed preference value in Group 2. In both cases, a small p-value suggests that the preference in Group 2 is unlikely to have been generated by the same distribution and hence underlying processes as Group 1. A significance level can be specified to formally determine whether a change in interaction preference between two groups of networks is deemed significant: we consider a p-value < 0.01 to be significant.

For an interaction observed in Group 2 but not Group 1 (a switch, see Supplementary Note 3) the significance of a preference in Group 2 can still be measured by a p-value, but it is calculated in a slightly different way from above. First, a synthetic distribution of interaction preferences is generated following the above four steps, but using data from Group 2 rather than Group 1. We then test whether the distribution associated with the interaction in Group 2 is significantly different from the mass action preference value of one. If the mean of the distribution is greater than one, then the p-value is equal to the fraction of values in the distribution lower than one, i.e., the p-value is the probability of observing a preference value in Group 2 that is lower than mass action. But if the mean of the distribution is lower than one, then the p-value is equal to the fraction of values in the distribution greater than one, i.e., the p-value is the probability of observing a preference value in Group 2 that is higher than mass action. As above, we consider a p-value < 0.01 to be significant.

A summary of changes in interaction preferences for our data sets is given in Supplementary Table 2. We find that incumbent interactions, those in both groups of networks, are more likely to significantly change in preference compared to switches. Among incumbent interactions, there are more significant increases in preference than decreases. There is no corresponding pattern with switches.

Group-level deviation of weighted network structure from mass action

For a group of networks, we quantify the effect of individual species' deviations from mass action on network structure by comparing the likelihood with $\alpha = \hat{\alpha}$ and $\beta = \hat{\beta}$—the best possible likelihood consistent with mass action—to the *overall* best likelihood that could be obtained in our

approach. The overall best likelihood results when probabilities in Eqn S3 are set as

$$p_{ijk} \propto \gamma_{ij}\hat{x}_i\hat{x}_j = \frac{\gamma_{ij}\hat{x}_i\hat{x}_j}{\sum_{ij}\gamma_{ij}\hat{x}_i\hat{x}_j} \tag{S8}$$

(recall that interaction preferences are averaged across networks indexed by field site k). In this expression, the product of maximum likelihood estimate abundances is scaled by the preference, γ_{ij}, for each interaction. Because γ_{ij} is derived from the set of recorded counts (see Eqns S6 and S7), and likelihood is calculated with respect to the set of recorded counts, Eqn S8 results in the best possible likelihood. Or put another way: γ_{ij} represents the best possible scaling of relative species abundances to match recorded counts, and, as such, results in the best possible likelihood. Thus, the group-level deviation of weighted network structure from mass action is linked to γ_{ij}, and, more specifically, the extent to which non-zero interaction preferences differ from their mass action value of one.

Denote the best possible likelihood consistent with mass action as $L_{\text{ma}} = L(\hat{\alpha}, \hat{\beta}, \hat{x}_i, \hat{x}_j | B_{ijk})$ and its corresponding log-likelihood as $\mathcal{L}_{\text{ma}} = \ln(L_{\text{ma}})$; and the overall best log-likelihood as $\mathcal{L}_{\text{best}} = \ln(L_{\text{best}})$. Group-level deviation from mass action can then be quantified as

$$\mathcal{B} = \frac{\mathcal{L}_{\text{ma}} - \mathcal{L}_{\text{best}}}{\mathcal{L}_{\text{ma}}} \tag{S9}$$

which ranges [0,1]. If $\mathcal{B} = 0$, then $\mathcal{L}_{\text{best}} = \mathcal{L}_{\text{ma}}$ and weighted network structure cannot be explained by anything additional to mass action. (Note that this is not the same as weighted network structure being completely explained by mass action, which would require $\mathcal{L}_{\text{ma}} = 0$.) If $\mathcal{B} = 1$, then $\mathcal{L}_{\text{best}} = 0$ and the preferences of γ_{ij} completely explain weighted network structure at a regional scale. In practice, $\mathcal{L}_{\text{best}}$ is larger than $\mathcal{L}_{\text{ma}}$ and both are non-zero. So, remembering that log-likelihoods are negative, the more of network structure that interaction preferences (γ_{ij}) can explain that mass

action cannot, the larger the value of $\mathcal{B}$, and the more of weighted network structure that can be attributed to deviation from mass action. A summary of group-level deviation of weighted network structure from mass action for our data sets is given in Supplementary Table 1.

As an aside, with some groups of networks it is important to use $\alpha = \hat{\alpha}$ and $\beta = \hat{\beta}$ and not a simpler analysis with $\alpha = 1$ and $\beta = 1$. Otherwise, changes in weighted network structure that could be explained by changes in relative species abundance would be assigned to changes in interaction preference, which is undesirable. The effect of maximum likelihood estimate parameters for explaining counts in a group of networks can be assessed by comparing the likelihood with $\alpha = \hat{\alpha}$ and $\beta = \hat{\beta}$ to the likelihood with $\alpha = 1$ and $\beta = 1$ (Supplementary Table 1). If likelihoods are similar, then the maximum likelihood distribution of estimated host and parasitoid species' abundances are similar to those for the simpler version of mass action. In which case, the maximum likelihood estimate values of α and β are unimportant. However, if the likelihood with $\alpha = \hat{\alpha}$ and $\beta = \hat{\beta}$ is notably higher than with $\alpha = 1$ and $\beta = 1$, then one or both distributions of estimated abundances are notably stretched or compressed compared to that for the simpler version of mass action. In this case, it is important to use $\alpha = \hat{\alpha}$ and $\beta = \hat{\beta}$.

Other probability distributions for the likelihood function

There are other probability distributions besides the multinomial distribution for computing likelihoods. Below, we discuss the application of six other probability distributions: binomial, Poisson binomial, Poisson, compound Poisson, hypergeometric and multivariate hypergeometric. We pay particular attention to the binomial distribution, which will be especially useful in future studies as more and better data become available.

Binomial distribution

The binomial distribution is useful for describing individual interactions rather than the inherently multiple-interaction perspective of the multinomial distribution. In the context of weighted interaction networks, the number of trials n is most naturally understood as the number of encounters between a particular pair of species at a given field site, i.e., $n = n_{ijk}$, and the probability that an encounter leads to an interaction count (a "successful outcome") is given by a distinct probability $q = q_{ijk}$. With this formulation, the likelihood function for the binomial distribution is

$$L_{\text{binomial}}(n_{ijk}, q_{ijk}|B_{ijk}) = P(B_{ijk}|n_{ijk}, q_{ijk}) = \frac{n_{ijk}!}{B_{ijk}!(n_{ijk} - B_{ijk})!} q_{ijk}^{B_{ijk}} (1 - q_{ijk})^{n_{ijk} - B_{ijk}} \quad \text{(S10)}$$

where the recorded number of counts B_{ijk} is used for the total number of "successes."

A tricky question given currently available data is how to determine appropriate values for n_{ijk} and q_{ijk}. Regarding n_{ijk}, it is not advisable to use effective abundances for two reasons. First, the product $n_{ijk} \sim \hat{x}_i \hat{x}_j$ is not guaranteed to give an integer. Second, an effective abundance is not guaranteed to reflect the general abundance of a species, rather, it represents the component of abundance that directly contributes to counts in an interaction network. Furthermore, if $n_{ijk} = f(\hat{x}_i, \hat{x}_j)$ then it is not clear that it would be reasonable to use $q_{ijk} = f(\gamma_{ij})$, so it would be necessary to devise another practical way of determining the "preference" of an interaction.

We suggest that the binomial distribution is most appropriate when independent measurements of species abundance are available in addition to interaction data. The binomial distribution then offers a nice two-step approach to calculating likelihoods. Let us represent independent abundance estimates by X_i for hosts and X_j for parasitoids (we do not include a k-index to maintain

consistency with effective abundances, but the described approach is clearly possible if independent estimates are available at the level of individual field sites). In Step I, we can use independent abundances to model the expected number of encounters between each pair of species, i.e., $n_{ijk} \sim X_i X_j$, which could also be modified by an additional multiplicative factor to reflect differences among interactions due to environmental or foraging effects. In Step II, we can use interaction data to determine the conversion probability for an interaction count given an encounter, i.e., $q_{ijk} = f(\gamma_{ij})$ or $q_{ijk} = f(\gamma_{ijk})$ or something similar. The new interpretation of "preference" then represents whether an individual parasitoid "chooses" to parasitise the host following an encounter, and/or, following oviposition, to what extent host quality, natural immune response and internal competition following multiple infections affects the chances of an encounter resulting in a successful parasitism event. (With plant-pollinator visitation networks, Step II would capture whether or not a pollinator "chooses" to feed on a particular plant species.)

Framed in this way, it becomes clear that our current approach using interaction preferences and the multinomial distribution essentially condenses Steps I and II into a single step, which is necessary given the lack of data on independent species abundances. Despite this correspondence between the binomial and multinomial distributions, the two probability distributions represent very different stochastic processes with very different underlying assumptions. The binomial distribution represents the probability of recording a successful parasitism event given a host-parasitoid encounter in the field and, as such, describes the probability that an individual parasitoid attacks a host and lays an egg that successfully develops into an adult. It is therefore most appropriate if adult parasitoids are expected to attempt oviposition on the first host they encounter, irrespective of community composition. By contrast, the multinomial distribution represents the probability that a

parasitoid picks a given host (leading to a successful parasitism event), conditioned on information about other potential hosts in the community. It is therefore able to directly account for higher-order processes such as active foraging or host hiding. Because there is a distinct multinomial distribution for each community composition, the raw probabilities used to compute likelihood are most useful when community composition remains relatively stable. For example, the loss of a single host species from a community may not matter much, especially if the total number of potential host individuals to be parasitised changes very little, but the multinomial distribution gradually loses relevance as further species are lost. In the extreme case of a single pair of host and parasitoid species, the multinomial distribution is no longer informative whereas the binomial distribution is still valid. Our four data sets each contained similar sets of species in both modified and unmodified habitat types, so the multinomial distribution was appropriate and allowed us to directly test for changes in host selectivity. However, it will be important to check that the assumptions underlying both the binomial and multinomial distributions are acceptable with new data sets, particularly those involving other types of ecological network.

Poisson binomial distribution

The Poisson binomial distribution is the probability distribution of a sum of independent Bernoulli trials that are not necessarily identically distributed. It is the probability distribution of the number of successes in a sequence of n independent yes/no experiments with success probabilities $q_1, q_2, \ldots, q_n$ (the ordinary binomial distribution is the special case when $q_1 = q_2 = \ldots = q_n$). There is a subtle point of difference when considering multiple interactions with the Poisson binomial distribution compared to using the binomial distribution: the likelihood function associated

with the Poisson binomial distribution would measure the ability of a model to generate the same number of total counts in an empirical matrix (i.e., $\sum_{ijk} B_{ijk}$), rather than the likelihood of each interaction individually.

Poisson distribution

As data are matrices of interaction counts, we could consider using a Poisson distribution. The likelihood function for the Poisson distribution is

$$L_{\text{Poisson}}(\lambda_{ijk}|B_{ijk}) = P(B_{ijk}|\lambda_{ijk}) = \frac{\lambda_{ijk}^{B_{ijk}} e^{-\lambda_{ijk}}}{B_{ijk}!} \tag{S11}$$

where λ_{ijk} is the expected number of counts for the interaction between host species i and parasitoid species j at field site k. In using the Poisson distribution, however, we would lose many of the links to ecological processes and mechanisms afforded by the multinomial and binomial distributions.

Compound Poisson distribution

Similar to the relationship between the binomial distribution and the Poisson binomial distribution, the compound Poisson distribution is a potential extension of the Poisson distribution to multiple interactions in a community. We suggest that for the current purpose the compound Poisson distribution suffers from the same disadvantages as the regular Poisson distribution.

Hypergeometric distribution

The hypergeometric distribution is a discrete probability distribution that describes the probability of k successes in n draws, *without* replacement, from a finite population of size N that contains

exactly K successes, and each draw is either a success or a failure. By contrast, the binomial distribution describes the probability of k successes in n draws *with* replacement.

For our system, computing likelihoods using the hypergeometric distribution requires an approximation for the total number of encounters between two species, e.g., $N_{ijk} \propto X_i X_j$; where X_i and X_j are independent abundance estimates for hosts and parasitoids, respectively. And an approximation for the total number of success, e.g., $K_{ijk} \propto \gamma_{ij} \hat{x}_i \hat{x}_j$. The likelihood function for the hypergeometric distribution then compares these N_{ijk} and K_{ijk} to the observed number of successes $k_{ijk} = B_{ijk}$ given some sampling parameter n_{ijk}. Framed in this way, it is clear that using the hypergeometric distribution to compute likelihoods is currently not feasible with available data. It is, however, potentially useful in future work to investigate the effect of sampling effort on the structure of weighted interaction networks. For example, once N_{ijk} and K_{ijk} have been adequately parameterised then one could explore how k_{ijk} varies under different n_{ijk}.

Multivariate hypergeometric distribution

The multivariate hypergeometric distribution is the without-replacement equivalent to the multinomial distribution (which assumes replacement).

The issue of potential and realised niche

In the main text, we suggest that laboratory experiments to measure “inherent” preferences would also help untangle the issue of potential and realised niche. In this context, “inherent” preferences can be considered as analogous to the potential rather than realised niche, but at the population level

rather than the individual level. Although in this study we have focused on predicting the effects of habitat modification on the feeding preferences of species, it is worth emphasising that even in undisturbed systems we cannot be certain of measuring what could be considered underlying or “inherent” preferences. This is because the feeding interactions observed in these systems may be a subset of those that could be achieved in the absence of competitors: we are likely to be observing the realised feeding niches of these species, and their fundamental feeding niches may be broader (or differ in magnitude) from those observed under natural conditions. The only way to fully resolve the relationship between potential and realised niche would be a series of manipulative experiments where preferences are assessed across all sets of hosts for each parasitoid species, in isolation from competition. This is not feasible in most systems. But in the absence of data on true preferences, although imperfect, our version of preferences are likely to be at least broadly accurate and may be useful as a first step at predicting changes to weighted network structure.

SUPPLEMENTARY NOTE 3: Switches

We define a *switch*[13] as an interaction that is known to be possible in a novel environment but has not been recorded in existing network data. In practice, a switch is an interaction that is observed in a group of networks representing a novel environment (the test group), but not observed in the group of networks used to calibrate a model (the calibration group). By contrast, we refer to an interaction that is observed in both groups as an incumbent interaction. We consider two possibilities for switches: i) one or both species associated with a switch are so rare as to make observing a successful parasitism event highly unlikely, yet the non-observation is nevertheless consistent with mass action; or ii) the interaction is not observed because it is a highly less-preferred interaction. As a switch is an interaction that, by definition, is not observed in the group of networks used to calibrate a model, we cannot infer a preference value using the methods described above. Therefore, if we want to use information from one group of networks to predict counts in another group, then we must devise additional methods for estimating interaction preferences for switches. For the first case we assume that switches follow mass action, while for the second case we use a binomial method to assign interaction preferences to switches with a value between zero and one.

With our data sets, mass action switches consistently performed better than binomial method switches with the alternative preferences model (Supplementary Table 3). As such, we modelled switches as following mass action in the correlated preferences and specified preferences models. Switches are not considered in the aggregate counts model; they are inherently assumed to follow mass action in the random encounter model; and they do not require special modelling in the complete characterisation model. Although models incorporating mass action switches performed better than binomial method switches, it is worth noting that the interaction preferences of some

switches still deviated significantly from mass action in all but one pair of calibration and test groups (Supplementary Table 2).

Mass action switches

The simplest way of modelling switches is to assume they follow mass action and should therefore be assigned a preference value equal to one. This implicitly assumes two, not necessarily mutually exclusive, possibilities: that one or both species associated with a switch were too rare for the interaction to be observed at field sites in the calibration group and species' abundances were higher when the interaction was observed at field sites in the test group; and/or habitat complexity interfered with encounters between host and parasitoid (e.g., through appreciable host hiding or complicated parasitoid foraging environment), leading to non-observation at field sites in the calibration group, and the environment was different when the interaction was observed at field sites in the test group. After identifying an interaction between host species i and parasitoid species j as a switch, we model it as following mass action by setting $\gamma_{ij} = 1$, when otherwise it would have interaction preference equal to zero (see Eqns S6 and S7).

Estimating interaction preferences of switches using the binomial method

If we assume that switches are somehow inherently less-preferred interactions, then they must have interaction preference $\gamma_{ij} < 1$. In which case, we estimate values for γ_{ij} in the range [0,1] based on the estimated abundances of the two species involved in the interaction. We require that interactions between abundant species be assigned smaller preference values than interactions between rarer species. This is because, in general, we expect a greater number of random encounters and hence successful parasitism events between abundant species, so the non-observation of an interaction in

this case should be penalised more strongly by assigning a lower value for interaction preference.

Assume that the number of counts for a switch follows a binomial distribution:

$$K \sim \mathrm{B}(n, p) \tag{S12}$$

where n is the number of expected encounters (trials) and p is the probability of observing a successful parasitism event (count). The probability of observing exactly k successes in n trials is given by the probability mass function:

$$\Pr(K = k) = \binom{n}{k} p^k (1-p)^{n-k} \tag{S13}$$

As we are interested in the case in which no count is recorded, we set $k = 0$. We also set $\Pr(K = 0) = \frac{1}{2}$, which assumes that if only one trial is considered then there is a fifty-fifty chance of recording a count. The probability of observing a successful parasitism event is then

$$p = 1 - 2^{-\frac{1}{n}} \tag{S14}$$

which returns values in the range [0,1], with the larger is n, the lower is p.

The host and parasitoid species associated with a switch are often involved in interactions with other species. If counts are recorded for those interactions, then we can obtain maximum likelihood estimates for their relative abundances, $\hat{x}_i$ and $\hat{x}_j$ (Eqn S5). The number of expected encounters between two species is proportional to the product of estimated abundances, so, substituting for n in Eqn S14, we estimate the interaction preference of switches using the binomial method as

$$\gamma_{ij} = 1 - 2^{-\frac{1}{\hat{x}_i \hat{x}_j}} \tag{S15}$$

which has range [0,1], as above. Because its maximum value is one, all switches are considered to be inherently less-preferred interactions, as required, and the more we expected to observe an interaction but do not, the lower the interaction preference of a switch.

Determining whether an observed number of switches is unexpectedly high or low

In addition to modelling switches, we also determine the significance of the number of interactions that we categorise as switches. To illustrate our method, consider two groups of networks containing a common subset of host and parasitoid species: Group 1 and Group 2. There will be a particular number of switches in Group 1: $\mathcal{S}_1$ (interactions observed in Group 1 but not Group 2); and likely a different number of switches in Group 2: $\mathcal{S}_2$ (interactions observed in Group 2 but not Group 1). We can determine whether an observed number of switches is unexpectedly high or low by comparing $\mathcal{S}_1$ and $\mathcal{S}_2$ to distributions generated by repeatedly partitioning the complete set of networks into two groups at random.

Assume there are N_1 networks in Group 1 and N_2 in Group 2. Partition, at random, the $N_1 + N_2$ networks into two new groups containing the same number of networks as in the original groups. Then calculate the number of switches in each of the new groups. Repeating this process multiple times results in a distribution of values: $\mathcal{S}'_1$ and $\mathcal{S}'_2$ (we performed 10,000 realisations). By comparing the observed number of switches to its corresponding distribution, we can calculate the probability of obtaining, relative to random partitioning of networks into groups, a greater number of switches than that observed:

$$p = P(\mathcal{S}' > \mathcal{S}) \tag{S16}$$

If $p < 0.05$ then the observed number of switches is much higher than expected owing to the

original grouping of networks; while if $p > 0.95$ then the observed number of switches is much lower than expected.

Results for our data sets are given in Supplementary Table 2. In general, we observe significantly large numbers of switches in open compared to forested habitat types, and significantly large numbers of switches in habitat types with high consumer-resource ratio compared to low.

SUPPLEMENTARY NOTE 4: Assortativity and disassortativity

Assortativity is a tendency for nodes in a network to connect to other nodes that have similar properties; with disassortativity, nodes connect to other nodes that have dissimilar properties[14–16]. The most commonly considered node property is degree, which is the total number of (binary) edges incident to a node. In an assortative network, nodes with high degree tend to connect to nodes with high degree, and nodes with low degree tend to connect to nodes with low degree. In a disassortative network, nodes with high degree tend to connect to nodes with low degree, and *vice versa*. There is a natural extension of assortativity and disassortativity to weighted networks[16]. With weighted assortativity, edges with larger weight (however defined) contribute more to determining assortativity than do edges with smaller weight (and similarly with weighted disassortativity).

Binary and weighted assortativity and disassortativity are node-centric network properties. That is, they describe a pattern of how nodes connect to other nodes in a given network. For ecological networks, assortativity indicates that generalist species tend to interact with other generalists (and specialists with specialists), whereas disassortativity indicates that generalists tend to interact with specialists. But this node-centric approach may not always be appropriate. Here we introduce an edge-centric version of weighted assortativity and disassortativity that we refer to as *interaction assortativity* and *interaction disassortativity*, respectively. Interaction assortativity indicates that high-weight interactions are incident to species involved in other, high-weight interactions, and low-weight interactions are incident to species involved in other, low-weight interactions. Interaction disassortativity indicates that high-weight interactions are incident to species involved in other, low-weight interactions, and *vice versa*.

In an interaction-assortative network in which edge weights are now interaction preferences (γ_{ij}, Eqn S7), some host species are involved in multiple interactions that are at the higher range of preferences for the community. By contrast, in an interaction-disassortative network, host species with at least one interaction at the higher range of preferences tend to be involved in other interactions that are at the lower range of preferences. Therefore, there are clear differences in the level of inter-specific competition between interaction-assortative and interaction-disassortative networks.

Binary networks

One way to determine the degree correlation in a network is to examine how the average degree of nearest neighbours varies with node degree[14]. (An alternative method is the assortativity coefficient, which is defined as the Pearson correlation coefficient of degree between pairs of connected nodes[15].) The average nearest-neighbour degree for node i is

$$k_{\text{nn},i} = \frac{\sum_j a_{ij} k_j}{\sum_j a_{ij}} \tag{S17}$$

where $a_{ij} = 1$ if there is a (binary) interaction between nodes i and j, and zero otherwise, and k_j is the degree of node j. At the network level, the average degree of nearest neighbours can be expressed as

$$k_{\text{nn}}(k) = \sum_{k'} k' P(k'|k) \tag{S18}$$

where $P(k'|k)$ is the conditional probability that a node with degree k is connected to a node with degree k'. If there are no degree correlations then $P(k'|k)$ does not depend on k and therefore $k_{\text{nn}}(k) =$ constant. If $k_{\text{nn}}(k)$ is an increasing function of k then high-degree nodes tend to connect to other high-degree nodes and the network is assortative. But if $k_{\text{nn}}(k)$ is a decreasing function of k then high-degree nodes tend to connect to low-degree nodes and the network is disassortative.

Weighted networks

The strength of a node can be defined in terms of the total weight of its edges:

$$s_i = \sum_j a_{ij} w_{ij} \tag{S19}$$

where, as above, $a_{ij} = 1$ if there is a (binary) interaction between nodes i and j, and zero otherwise, and w_{ij} is the weight of the edge. The weighted average nearest-neighbours degree is defined as[16]

$$k^{\text{w}}_{\text{nn},i} = \frac{1}{s_i} \sum_j a_{ij} w_{ij} k_j \tag{S20}$$

where k_j is the (binary) degree of node j. This definition implies that $k^{\text{w}}_{\text{nn},i} > k_{\text{nn},i}$ if edges with larger weight connect to nodes with larger degree, and $k^{\text{w}}_{\text{nn},i} < k_{\text{nn},i}$ if edges with larger weight connect to nodes with smaller degree. The function $k^{\text{w}}_{\text{nn},i}$, similar to Eqn S18, indicates the weighted assortativity or disassortativity of a network.

Weighted bipartite networks and interaction assortativity and disassortativity

Here we extend the idea of assortativity and disassortativity to weighted bipartite networks by moving from a node-centric to edge-centric approach. First, we calculate the average weight of each edge's nearest-neighbour edges. Then we determine how the average weight of nearest-neighbour edges varies with edge weight:

$$w_{\text{nn}}(w) = \sum_{w'} w' P(w'|w) \tag{S21}$$

where, similar to Eqn S18, $P(w'|w)$ is the conditional probability that an edge with weight w is a nearest neighbour of an edge with weight w'. If there are no edge-weight correlations then $P(w'|w)$ does not depend on w and therefore $w_{\text{nn}}(w) =$ constant. If $w_{\text{nn}}(w)$ is an increasing function of w then high-weight edges tend to be neighbours of other high-weight edges and the network is

interaction assortative. But if $w_{\text{nn}}(w)$ is a decreasing function of w then high-weight edges tend to be neighbours of low-weight edges and the network is interaction disassortative.

We consider three options for calculating nearest-neighbour edges in host-parasitoid (and similar) interaction networks: i) host based, in which interactions involving the same host species are nearest neighbours; ii) parasitoid based, in which interactions involving the same parasitoid species are nearest neighbours; and iii) both host and parasitoid based, in which interactions involving either the same host or parasitoid species are nearest neighbours.

With host-based nearest neighbours, the average nearest-neighbour weight for an interaction between host species $i = i'$ and parasitoid species $j = j'$ is

$$w^{\text{host}}_{\text{nn},i=i',j=j'} = \frac{\sum_j \ln(w_{i=i',j}) - \ln(w_{i=i',j=j'})}{\sum_j a_{i=i',j} - 1} \tag{S22}$$

where $a_{i=i',j} = 1$ if there is a (binary) interaction between host species $i = i'$ and parasitoid species j and $a_{i=i',j} = 0$, otherwise. As we are interested in the average weight of nearest-neighbour edges, we exclude the weight of the focal edge, $w_{i=i',j=j'}$, when calculating the average.

With parasitoid-based nearest neighbours, the average nearest-neighbour weight for an interaction between host species $i = i'$ and parasitoid species $j = j'$ is

$$w^{\text{parasitoid}}_{\text{nn},i=i',j=j'} = \frac{\sum_i \ln(w_{i,j=j'}) - \ln(w_{i=i',j=j'})}{\sum_i a_{i,j=j'} - 1} \tag{S23}$$

where $a_{i,j=j'} = 1$ if there is a (binary) interaction between host species i and parasitoid species $j = j'$ and $a_{i,j=j'} = 0$, otherwise.

With both host- and parasitoid-based nearest neighbours, the average nearest-neighbour weight

for an interaction between host species $i = i'$ and parasitoid species $j = j'$ is

$$w^{\text{both}}_{\text{nn},i=i',j=j'} = \frac{\sum_j \ln(w_{i=i',j}) + \sum_i \ln(w_{i,j=j'}) - 2\ln(w_{i=i',j=j'})}{\sum_j a_{i=i',j} + \sum_i a_{i,j=j'} - 2} \tag{S24}$$

Following the reasoning associated with Eqn S21, we can determine interaction assortativity or disassortativity for a weighted bipartite network using all three definitions of nearest-neighbour edges. But because we are interested in competition between parasitoid species and the effect on interaction preferences and hosts, we focus on the host-based definition of nearest neighbours (Eqn S22).

Effect of maintaining parasitoid rank-order interaction preferences on edge-weight correlations

For each parasitoid species in a group of networks, we can identify its rank order of interaction preferences using γ_{ij} (Eqn S7). The host species associated with the largest preference among its set of interactions is its top-ranked interaction partner, the smallest preference is its bottom-ranked interaction partner, and its remaining host species are sorted in between. This particular rank order of interaction preferences may change when considering a different group of networks containing the same parasitoid and host species. If networks are grouped according to habitat type, as here, then a change in rank-order preferences could reflect a change in, for example, host selectivity by parasitoids that is mediated by habitat complexity. In which case, there could be an effect on inter-specific competition that influences the group-level pattern in edge-weight correlations (measured using Eqn S21).

Consider two groups of networks, Group 1 and Group 2, and we want to know what effect

rank-order interaction preferences in Group 1 has on edge-weight correlations in Group 2. First, we derive a preference matrix (with entries γ_{ij}, Eqn S7) from recorded counts in Group 1 (we assume that switches follow mass action and are therefore assigned preference value equal to one, see Supplementary Note 3). From this preference matrix we obtain rank-order preferences for each parasitoid species in Group 1. Then we derive a second preference matrix from recorded counts in Group 2. For each parasitoid species in this second preference matrix, we reorder interaction preferences according to the rank order in Group 1 to give a third, synthetic, preference matrix. (If there are tied rank-order preferences, such as when a parasitoid species in Group 1 is involved in more than one switch, then multiple reorderings are possible, in which case we select one of the possible reorderings at random. This means that many synthetic preference matrices are consistent with the rank-order preferences from Group 1. As such, we generated 10,000 synthetic preference matrices for analysis.)

The three preference matrices—derived from recorded counts in Group 1, recorded counts in Group 2, and recorded counts in Group 2 but reordered according to rank-order preferences in Group 1—have the same topological (binary) structure but potentially very different weighted structure. We can compare how the average weight of nearest-neighbour edges varies with edge weight (Eqn S21) for the three preference matrices. We consider two comparisons. The first comparison is between the preference matrix derived from recorded counts in Group 2 and when interaction preferences are reordered according to rank-order preferences in Group 1; in this case, the set of preference values is the same but rank-order preferences are different. So if networks are grouped according to habitat type, then with this comparison we are testing the effect of habitat complexity on rank-order preferences and, consequently, edge-weight correlations (which indicates

whether a network is interaction assortative or disassortative, see above). The second comparison is between the preference matrix derived from recorded counts in Group 1 and the preference matrix derived from recorded counts in Group 2 but reordered according to rank-order preferences in Group 1; in this case, rank-order preferences are the same but preference values are different. If edge-weight correlations are similar in the second comparison but different in the first comparison, then it would suggest that habitat complexity mediates rank-order interaction preferences, with any changes in rank-order interaction preferences observable as changes in edge-weight correlations.

With our data sets, the pattern of interactions onto hosts is interaction disassortative in open habitats, but there is no significant correlation in forested habitats (Supplementary Fig. 1). This suggests that high amounts of habitat complicates host finding by parasitoids, which results in a systematic response of communities to changes in habitat complexity. When maintaining rank-order interaction preferences from habitats with no correlation pattern, we still observed no pattern with a new set of interaction preferences, even in habitats where otherwise we observed significant interaction disassortativity. When maintaining rank-order interaction preferences from habitats with significant interaction disassortativity, we still observed interaction disassortativity with a new set of interaction preferences, even in habitats where otherwise we observed no correlation pattern. Therefore, at the species level, habitat complexity appears to mediate the rank-order interaction preferences of parasitoids. This finding motivates the correlated preferences model.

SUPPLEMENTARY NOTE 5: Models and prediction

We developed a series of models for predicting weighted network structure at new field sites in a novel environment. We assessed the performance of models by selecting pairs of groups from the same data set: models were parameterised using data from the calibration group and predictions were tested using recorded counts from the test group, which represents the novel environment. Let us denote variables in the calibration group by B_{ijk}^{cal}, $\hat{x}_i^{\text{cal}}$, $\hat{x}_j^{\text{cal}}$ and γ_{ij}^{cal}; and variables in the test group by B'_{ijk}, $\hat{x}'_i$, $\hat{x}'_j$ and γ'_{ij}. Each model generates probabilities p_{ijk} that are compared to B'_{ijk} using Eqn S3 to calculate log-likelihoods, which we use to measure and compare model performance at individual field sites (Eqn S25) and at the group level (Eqn S26). The seven models we considered and their data requirements for calibration are summarised in Table 1 of the main text.

Null model with uniform interaction frequencies

All interactions have the same probability, $p_{ijk} = \frac{1}{\sum_{ijk} a_{ijk}}$; where $a_{ijk} = 1$ if $B'_{ijk} > 0$, and zero otherwise, i.e., $\sum_{ijk} a_{ijk}$ is the number of non-forbidden interactions recorded at a field site (ignoring counts). This model assumes that knowing binary network structure is sufficient to predict weighted network structure at a field site.

Aggregate counts model

Probabilities are set proportional to the number of recorded counts summed across networks from different field sites in the calibration group: $p_{ijk} = \frac{\sum_k B_{ijk}^{\text{cal}}}{\sum_{ijk} B_{ijk}^{\text{cal}}}$. This model assumes that known interaction frequencies at a group level (i.e., counts summed across networks sampled at different field sites) are informative at new field sites in novel environments.

Random encounter model

Probabilities are set proportional to the product of effective abundances of host and parasitoid species in the novel environment: $p_{ijk} = \frac{\hat{x}'_i \hat{x}'_j}{\sum_{ij} \hat{x}'_i \hat{x}'_j}$; recall that effective abundances are assumed to hold across all field sites in a group, which is why there is no k-index on the right-hand side of the expression for p_{ijk}. Notice that this model assumes only mass action and the likelihood function reduces to Eqn S4.

Alternative preferences model

Probabilities are set proportional to the product of an existing preference matrix $\gamma_{ij}^{\text{alt}} = \gamma_{ij}^{\text{cal}}$ and effective abundances in the novel environment: $p_{ijk} = \frac{\gamma_{ij}^{\text{alt}} \hat{x}'_i \hat{x}'_j}{\sum_{ij} \gamma_{ij}^{\text{alt}} \hat{x}'_i \hat{x}'_j}$. For switches (interactions known to be possible but with no entry in γ_{ij}^{alt}), we considered two possibilities: i) switches follow mass action and we set $\gamma_{ij}^{\text{alt}} = 1$; or ii) switches are inherently less-preferred interactions and we set $\gamma_{ij}^{\text{alt}} = 1 - 2^{-\frac{1}{\hat{x}_i^{\text{cal}} \hat{x}_j^{\text{cal}}}}$, which returns values between zero and one, in inverse proportion to the product of effective abundances in the calibration group. As discussed in Supplementary Note 3, mass action switches consistently led to better model performance.

Correlated preferences model

First, we obtain the column-wise rank order of interaction preferences in γ'_{ij}, i.e., host species are sorted and identified (first, second, third etc.) from highest-to-lowest interaction preference for each parasitoid species. This rank order represents a systematic pattern in interaction preferences that is identifiable with the novel environment (see Supplementary Note 4). We then reorder entries in γ_{ij}^{cal} (including mass action switches) according to the rank order in γ'_{ij} to obtain a new preference

matrix: $\gamma_{ij}^{\text{corr}}$. Probabilities are set as $p_{ijk} = \frac{(\gamma_{ij}^{\text{corr}})^{\hat{\delta}} \hat{x}'_i \hat{x}'_j}{\sum_{ij} (\gamma_{ij}^{\text{corr}})^{\hat{\delta}} \hat{x}'_i \hat{x}'_j}$; where $\hat{\delta}$ is a scaling parameter that is applied to each entry in the preference matrix and is set to its maximum likelihood estimate value (the model can also be used without the optimisation step, that is, with $\delta = 1$).

Specified preferences model

First, we determine the contribution of each interaction to log-likelihood by calculating Eqn S3 with B'_{ijk} and $p_{ijk} = \frac{\gamma'_{ij} \hat{x}'_i \hat{x}'_j}{\sum_{ij} \gamma'_{ij} \hat{x}'_i \hat{x}'_j}$ with all non-zero entries in γ'_{ij} set to one except the focal entry. We sort the log-likelihood contributions and identify the interactions above any obvious discontinuity (see Supplementary Figs. 2 and 3). We then replace—hardcode—the entries for influential interactions in γ_{ij}^{cal} (including mass action switches) with their corresponding values in γ'_{ij} to obtain a new preference matrix: $\gamma_{ij}^{\text{spec}}$. Probabilities are set as $p_{ijk} = \frac{\gamma_{ij}^{\text{spec}} \hat{x}'_i \hat{x}'_j}{\sum_{ij} \gamma_{ij}^{\text{spec}} \hat{x}'_i \hat{x}'_j}$. In this way, $\gamma_{ij}^{\text{spec}}$ is mainly based on data from the calibration group, except for a small fraction of interactions that make the biggest difference to likelihood and must be characterised individually in the novel environment. A variation of the specified preferences model has all non-zero entries in $\gamma_{ij}^{\text{spec}}$ set consistent with mass action and therefore with their preference values equal to one, except for the small fraction of hard-coded values: $\gamma_{ij}^{\text{spec2}}$. By comparing these two models we can test whether any useful information is contained in the preferences of interactions other than those that are hardcoded (Supplementary Table 3). The specified preferences and correlated preferences models can be combined by hardcoding entries for the influential interactions in $\gamma_{ij}^{\text{corr}}$ (see above) rather than γ_{ij}^{cal}.

Complete characterisation model

All interaction preferences must be characterised individually in the novel environment and so the relevant preference matrix is $\gamma_{ij}^{\text{complete}} = \gamma'_{ij}$. Probabilities are set as $p_{ijk} = \frac{\gamma_{ij}^{\text{complete}} \hat{x}'_i \hat{x}'_j}{\sum_{ij} \gamma_{ij}^{\text{complete}} \hat{x}'_i \hat{x}'_j}$. The model results in the best fit to data possible in our current approach and, by definition, returns the maximum model performance at the group level. It therefore represents an upper bound to predictions using our approach and its likelihood is discussed in Supplementary Note 2 (see Eqn S8).

Model performance at individual field sites

We measured the performance of model M at field site k as

$$\mathcal{F}_{M,k} = \frac{\mathcal{L}_{\text{null},k} - \mathcal{L}_{M,k}}{\mathcal{L}_{\text{null},k}} \tag{S25}$$

where the null model is described above and $\mathcal{L}_{\text{null},k}$ and $\mathcal{L}_{M,k}$ are log-likelihoods calculated using Eqn S3 with a single k-index. $\mathcal{F}_{M,k} = 1$ if model M completely explains the distribution of recorded interaction counts at field site k; $\mathcal{F}_{M,k} = 0$ if it performs the same as the null model; and $\mathcal{F}_{M,k} < 0$ if it performs worse than the null model.

Model performance at the group level

We measured the performance of model M at the group level as

$$\mathcal{R}_M = \frac{\mathcal{L}_{\text{re}} - \mathcal{L}_M}{\mathcal{L}_{\text{re}} - \mathcal{L}_{\text{complete}}} \tag{S26}$$

where the random encounter (re) and the complete characterisation (complete) models are described above, and $\mathcal{L}_{\text{re}}$, $\mathcal{L}_{\text{complete}}$ and $\mathcal{L}_M$ are log-likelihoods calculated using Eqn S3 for all field sites in a group of networks together, and, therefore, with multiple k-indices. $\mathcal{R}_M = 1$ if model M performs as well as the complete characterisation model; $\mathcal{R}_M = 0$ if it performs the same as the

random encounter model; and $\mathcal{R}_M < 0$ if it performs worse than the random encounter model. Log-likelihoods for the above models and the four host-parasitoid data sets are in Supplementary Table 3.

Targeted data collection of interactions between abundant species

As mentioned in the main text when discussing the specified preferences model, if the identity of interactions to target is not known in advance, then a good rule of thumb is to focus on interactions between the more abundant species. Here we present results for model performance at the group level (Eqn S26) when interactions between abundant species are hardcoded in the specified preferences model (rather than the interactions that contribute most to log-likelihood). We used each species' estimated abundance ($\hat{x}_i$ or $\hat{x}_j$) multiplied by the number of field sites at which it was recorded as present to determine the overall abundance of a species in a novel environment (in the test group).

For the Ecuador data set, we considered {forest, coffee, abandoned coffee} as the calibration group and {pasture, rice} as the test group. We selected the two most abundant host species (*Anthidium* sp. and *Pseudodynerus* sp.) and the two most abundant parasitoid species (*Chrysis* sp. and *Melittobia acasta*) in the test group (according to our measure of overall abundance, described above)—the same number of species involved in the previously hardcoded interactions—and hardcoded all four interactions between the four species in the specified preferences model, with non-hardcoded values being those from the alternative preferences model (i.e., calibrated with data from {forest, coffee, abandoned coffee} to determine a starting preference matrix). Hardcoding these four interactions (12% of the total number of distinct interactions in the test group) resulted

in $\mathcal{R}_M = 0.87$. In this case, the four abundant species were the same four species involved in the previously hardcoded interactions in the specified preferences model (Supplementary Fig. 3).

For the Indonesia data set, we considered {more forested} as the calibration group and {less forested} as the test group. We selected the two most abundant host species (*Auplopus levicarinatus* and *Rhynchium haemorrhoidale umeroater*) and the three most abundant parasitoid species (*Chrysis* sp.2; *Chrysis smaragdula*; and *Stilbum chrysochephalum*) in the test group (the same number of species involved in the previously hardcoded interactions, but not necessarily the same species) and hardcoded all six interactions (18% of the total number of distinct interactions) between the five species (using the same procedure as for Ecuador). These new hardcoded interactions included four of the previously hardcoded interactions but only resulted in $\mathcal{R}_M = 0.19$. However, when the five most abundant parasitoid species were selected, all six previously hardcoded interactions were included in the new analysis and model performance increased to $\mathcal{R}_M = 0.68$.

For the Swiss meadow data set, we considered {25m, 50m, 100m} as the calibration group and {ECA} as the test group. We selected the four most abundant host species (*Ancistrocerus gazella*; *Passaloecus gracilis*; *Trypoxylon figulus*; and *Trypoxylon* sp.) and the three most abundant parasitoid species (*Anthrax anthrax*; *Chrysis cyanea*; and *Melittobia acasta*) in the test group and hardcoded six interactions (15% of the total number of distinct interactions) between the seven species (not all of the 12 theoretically possible interactions were recorded in the test group). These new hardcoded interactions included four of the previously hardcoded interactions and resulted in $\mathcal{R}_M = 0.53$.

For the Swiss lowland data set, we considered {10 most forested from adjacent and con-

nected} as the calibration group and {isolated} as the test group. We selected the three most abundant host species (*Osmia bicornis* (rufa); *Trypoxylon figulus*; and *Trypoxylon* sp.) and the three most abundant parasitoid species (*Cacoxenus indagator*; *Melittobia acasta*; and *Trichodes alvearius*) in the test group and hardcoded six interactions (6% of the total number of distinct interactions) between the six species. These new hardcoded interactions included two of the previously hardcoded interactions and resulted in $\mathcal{R}_M = 0.39$.

Penalising for model complexity

Here we give a brief assessment of model performance at the group level in the context of AIC and BIC, which are two popular methods for model selection based on likelihood[17]. With AIC, model fit is balanced against model complexity by assigning a penalisation of one point of log-likelihood to each parameter (AIC $= 2k - 2\mathcal{L}_M$, where k is the number of parameters in model M). However, in many applications it is not always clear what should be considered a parameter in a model[18]. As a conservative assessment, we considered the effect of penalising one point of log-likelihood for each interaction that is hardcoded in the preference matrix associated with a model. At one extreme, this results in no complexity penalisation for the random encounter model. At the other, this results in a log-likelihood penalisation equal to the number of unique interactions in the test group of networks for the complete characterisation model (as all interactions must be characterised individually in a novel environment).

For the Ecuador data set when predicting between {forest, coffee, abandoned coffee} and {pasture, rice}, the difference in log-likelihood between the random encounter model and complete characterisation model was 334 (Supplementary Table 3); this is much larger than the relative

complexity penalisation, k, of 34 points of log-likelihood (see entry "E" in Supplementary Table 2) to the specified preferences model. For the Indonesia data set when predicting between {more forested} and {less forested}, the difference in log-likelihood was 15 with a penalisation difference of 35. For the Swiss meadow data set when predicting between {25m, 50m, 100m} and {ECA}, the difference in log-likelihood was 42 with a penalisation difference of 38. For the Swiss lowland data set when predicting between {10 most forested from adjacent and connected} and {isolated}, the difference in log-likelihood was 230 with a penalisation difference of 98. The difference in AIC between the two models was -600 (Ecuador), 40 (Indonesia), -8 (Swiss meadow) and -304 (Swiss lowland). So only with the Indonesia data set is it questionable to select the complete characterisation model over the random encounter model.

With BIC, we assigned a penalisation proportional to $\ln(\mathrm{H} \times \mathrm{P})$ points of likelihood for each hardcoded interaction, where H and P are the number of common host and parasitoid species, respectively, across calibration and test groups (see Supplementary Table 1). Specifically, BIC $=$ $k \ln(\mathrm{H} \times \mathrm{P}) - 2\mathcal{L}_M$. Clearly, BIC penalises model complexity more strongly than AIC. The difference in BIC between the random encounter and complete characterisation models was -486 (Ecuador), 149 (Indonesia), 123 (Swiss meadow) and 138 (Swiss lowland). So only with the Ecuador data set is it reasonable to select the complete characterisation model over the random encounter model.

Now consider penalising for the smaller number of hardcoded interactions in the specified preferences model. The difference in log-likelihood from the random encounter model was 290 (Ecuador), 11 (Indonesia), 29 (Swiss meadow) and 162 (Swiss lowland). The relative penalisa-

tion, k, with AIC was 3 (Ecuador), 6 (Indonesia), 6 (Swiss meadow) and 8 (Swiss lowland) (see Supplementary Fig. 3). The difference in AIC between the two models was -574 (Ecuador), -10 (Indonesia), -46 (Swiss meadow) and -308 (Swiss lowland). So it is reasonable to select the specified preferences model over the random encounter model for all data sets, according to AIC. The difference in BIC between the two models was -564 (Ecuador), 9 (Indonesia), -25 (Swiss meadow) and -272 (Swiss lowland). So only with the Indonesia data set is it questionable to select the specified preferences model over the random encounter model.

Consider the correlated preferences model for the above predictions. We assigned a one-parameter penalisation to this model (to account for $\hat{\delta}$). The difference in log-likelihood from the random encounter model was 145 (Ecuador), 6 (Indonesia), 9 (Swiss meadow) and 150 (Swiss lowland). The relative penalisation, k, with AIC was 1 for all data sets. The difference in AIC between the two models was -288 (Ecuador), -10 (Indonesia), -16 (Swiss meadow) and -352 (Swiss lowland). So it is reasonable to select the correlated preferences model over the random encounter model for all data sets, according to AIC. The difference in BIC between the two models was -284 (Ecuador), -7 (Indonesia), -12 (Swiss meadow) and -347 (Swiss lowland). So it is reasonable to select the correlated preferences model over the random encounter model for all data sets, according to BIC. This assumes no penalisation for reordering an existing preference matrix in the correlated preferences model.

In summary, for the Ecuador data set, the difference in AIC from the random encounter model was -288 (correlated preferences), -574 (specified preferences) and -600 (complete characterisation); which favours application of the complete characterisation model. The difference in

BIC was -284 (correlated preferences), -564 (specified preferences) and -486 (complete characterisation); which favours application of the specified preferences model. For the Indonesia data set, the difference in AIC was -10 (correlated preferences), -10 (specified preferences) and 40 (complete characterisation); which favours application of the correlated preferences and specified preferences models. The difference in BIC was -7 (correlated preferences), 9 (specified preferences) and 149 (complete characterisation); which favours application of the correlated preferences model. For the Swiss meadow data set, the difference in AIC was -16 (correlated preferences), -46 (specified preferences) and -8 (complete characterisation); which favours application of the specified preferences model. The difference in BIC was -12 (correlated preferences), -25 (specified preferences) and 123 (complete characterisation); which favours application of the specified preferences model. For the Swiss lowland data set, the difference in AIC was -352 (correlated preferences), -308 (specified preferences) and -304 (complete characterisation); which favours application of the correlated preferences model. The difference in BIC was -347 (correlated preferences), -272 (specified preferences) and 138 (complete characterisation); which favours application of the correlated preferences model. Of course, this model selection procedure does not take into account practical differences in sampling effort between models.

Supplementary References

1. Tylianakis, J.M., Tscharntke, T. & Lewis, O.T. Habitat modification alters the structure of tropical host-parasitoid food webs. *Nature* **445**, 202–205 (2007).

2. Klein, A.-M., Steffan-Dewenter, I. & Tscharntke, T. Rain forest promotes trophic interactions and diversity of trap-nesting hymenoptera in adjacent agroforestry. *J. Anim. Ecol.* **75**, 315–323 (2006).

3. Albrecht, M., Duelli, P., Schmid, B. & Müller, C.B. Interaction diversity within quantified insect food webs in restored and adjacent intensively managed meadows. *J. Anim. Ecol.* **76**, 1015–1025 (2007).

4. Coudrain, V., Schüepp, C., Herzog, F., Albrecht, M. & Entling, M. Habitat amount modulates the effect of patch isolation on host-parasitoid interactions. *Frontiers in Environmental Science* **2**, 27 (2014).

5. Peralta, G., Frost, C.M., Rand, T.A., Didham, R.K. & Tylianakis, J.M. Complementarity and redundancy of interactions enhance attack rates and spatial stability in host-parasitoid food webs. *Ecology* **95**, 1888–1896 (2014).

6. Montoya, D., Yallop, M.L. & Memmott, J. Functional group diversity increases with modularity in complex food webs. *Nat. Commun.* **6**, 7379 (2015).

7. Thompson, W.R. The utility of mathematical models in relation to work on biological control. *Ann. Appl. Biol.* **17**, 641–648 (1930).

8. Hassell, M.P. & Varley, C.G. New inductive model for insect parasites and its bearing on biological control. *Nature* **223**, 1133–1137 (1969).

9. Staniczenko, P.P.A., Kopp, J.C. & Allesina, S. The ghost of nestedness in ecological networks. *Nat. Commun.* **4**, 1931 (2013).

10. Holling, C.S. Resilience and stability of ecological systems. *Annu. Rev. Ecol. Evol. Syst.* **4**, 1–23 (1973).

11. R Core Team. *R: A Language and Environment for Statistical Computing*. R Foundation for Statistical Computing, Vienna, Austria (2014).

12. Vázquez, D., Chacoff, N.P. & Cagnolo, L. Evaluating multiple determinants of the structure of plant-animal mutualistic networks. *Ecology* **90**, 2039–2046 (2009).

13. Murdoch, W.W. Switching in general predators: experiments on prey specificity and stability of prey populations. *Ecol. Monogr.* **39**, 335–342 (1969).

14. Pastor-Satorras, R., Vázquez, A. & Vespignani, A. Dynamical and correlation properties of the internet. *Phys. Rev. Lett.* **87**, 258701 (2001).

15. Newman, M.E.J. Assortative mixing in networks. *Phys. Rev. Lett.* **89**, 208701 (2002).

16. Barrat, A., Barthélemy, M., Pastor-Satorras, P. & Vespignani, A. The architecture of complex weighted networks. *Proc. Natl. Acad. Sci. U.S.A.* **101**, 3747–3752 (2004).

17. Burnham, K.P. & Anderson, D.R. *Model Selection and Multimodel Inference: A Practical Information-Theoretic Approach* (Springer, 2002), second edn.

18. Staniczenko, P.P.A., Smith, M.J. & Allesina, S. Selecting food web models using normalized maximum likelihood. *Methods Ecol. Evol.* **5**, 551–562 (2014).
